# Supplementary material for: Content-rich biological network constructed by mining PubMed abstracts
Source: BMC Bioinformatics. 2004 Oct 8;5:147. doi: 10.1186/1471-2105-5-147 (PMC528731; doi:10.1186/1471-2105-5-147)
Supplement: Additional File 2 — The original results of the above study (non-essential files are deleted to keep the file size under the limit set by BMC bioinformatics). [file 1471-2105-5-147-S2.bz2 › chilibotAdditionalFile2/dip10/615ID9219685E10999/html/B2M_CD1D1.html]

 


 **B2M** and **CD1D1** 
  
Found 16 abstracts in PubMed, retrieved 10.  
 

 What does Google say? 
 PDF only 
| .edu only 

---

**Interactive relationship** (e.g. stimulation, inhibition, etc)

**Inhibitory relationship**- V1, V2, V4, and V5 lack a beta 2 microglobulin  [ **B2M** ]  binding site alpha3 domain, indicating the unstable presentation of the CD1d  [ **CD1D1** ]  molecule on the surface.  Ref: 11006091 Biochem Biophys Res Commun, 2000
**Neutral relationship**- In addition to MHC class I and II antigens, intestinal epithelia constitutively express the nonclassical MHC molecule CD1d  [ **CD1D1** ] , a transmembrane molecule with a short cytoplasmic tail expressed as a beta 2 microglobulin  [ **B2M** ]  associated 48 kDa glycoprotein and novel beta 2 microglobulin  [ **B2M** ]  independent 37 kDa nonglycosylated protein on intestinal epithelia.  Ref: 10570177 Proc Natl Acad Sci U S A, 1999
- We demonstrate that chaperone and foldase assisted refolding of denatured CD1d  [ **CD1D1** ]  molecules and beta 2 microglobulin  [ **B2M** ]  in the presence of synthetic lipids is a rapid method for the generation of functional and specific CD1d  [ **CD1D1** ]  tetramers, which unlike previously published protocols ensures isolation of CD1d  [ **CD1D1** ]  tetramers loaded with a single lipid species.  Ref: 11248072 Proc Natl Acad Sci U S A, 2001
- The biochemical structure of CD1d  [ **CD1D1** ]  on intestinal epithelial cells IECs exists in two forms a 37 kD nonglycosylated, beta 2 microglobulin  [ **B2M** ]  beta 2M independent and a 48 kD glycosylated, beta 2M dependent form.  Ref: 11296664 Keio J Med, 2001

**Non-interactive relationship** (e.g. studied together, co-existance, homology, etc.)

- Furthermore, CD4 T cells from CD1d  [ **CD1D1** ]  knockout KO B6 donor mice but not those from MHC I homozygous transgenic mice deficient for beta 2 microglobulin  [ **B2M** ]  KO B6 mice induced a colitis in RAG hosts.  Ref: 11238623 J Immunol, 2001
- In the present study to elucidate the recipient factor s that is responsible for the development of NK T cells, radiation bone marrow chimeras were established by using alymphoplasia mutant mice as a donor strain and either beta 2 microglobulin  [ **B2M** ]  knock out beta 2m or  **CD1d1**  mice as recipients, both of which lack CD1 molecules.  Ref: 10884971 Hokkaido Igaku Zasshi, 2000
- Recognition of infected targets by CD8 CTL was beta 2 microglobulin  [ **B2M** ]  and MHC class I dependent and was not CD1d  [ **CD1D1** ]  restricted.  Ref: 10861072 J Immunol, 2000
